# Clinical Outcomes Following Acute Residential Psychiatric Treatment in Transgender and Gender Diverse Adolescents

**DOI:** 10.1001/jamanetworkopen.2021.13637

**Published:** 2021-06-21

**Authors:** Marisa M. Silveri, Eleanor M. Schuttenberg, Kaya Schmandt, Elena R. Stein, Maya M. Rieselbach, Ariel Sternberg, Julia E. Cohen-Gilbert, Sabra L. Katz-Wise, Jennifer Urbano Blackford, Alexandra S. Potter, Mona P. Potter, Dana B. Sarvey, Chad M. McWhinnie, Jessica E. Feinberg, Kathryn D. Boger

**Affiliations:** 1 Neurodevelopmental Laboratory on Addictions and Mental Health, McLean Hospital, Belmont, Massachusetts; 2 Adolescent Acute Residential Treatment Program, McLean Hospital, Belmont, Massachusetts; 3 McLean Anxiety Mastery Program, McLean Hospital, Belmont, Massachusetts; 4 Department of Psychiatry, Harvard Medical School, Boston, Massachusetts; 5 Division of Adolescent/Young Adult Medicine, Boston Children’s Hospital, Boston, Massachusetts; 6 Department of Pediatrics, Harvard Medical School, Boston, Massachusetts; 7 Department of Social and Behavioral Sciences, Harvard T. H. Chan School of Public Health, Boston, Massachusetts; 8 Department of Psychiatry and Behavioral Sciences, Vanderbilt University Medical Center, Nashville, Tennessee; 9 Department of Psychiatry, University of Vermont, Burlington, Vermont

## Abstract

**Importance:**

Transgender and gender diverse (TGD) individuals, who have a gender identity that differs from their sex assigned at birth, are at increased risk of mental health problems, including depression, anxiety, self-injurious behavior, and suicidality, relative to cisgender peers.

**Objective:**

To examine mental health outcomes among TGD vs cisgender adolescents in residential treatment.

**Design, Setting, and Participants:**

This cohort study’s longitudinal design was used to compare groups at treatment entry and discharge, and 1-month postdischarge follow-up. The setting was an adolescent acute residential treatment program for psychiatric disorders. Participants were TGD or cisgender adolescents enrolled in the treatment program. Statistical analysis was performed October 2019 to March 2021.

**Exposure:**

Adolescents participated in a 2-week acute residential treatment program for psychiatric disorders.

**Main Outcomes and Measures:**

Primary outcomes were depressive (the Center for Epidemiologic Studies Depression Scale [CES-D]) and anxiety (the Multidimensional Anxiety Scale for Children [MASC]) symptoms, and emotional dysregulation (the Difficulties in Emotion Regulation Scale [DERS]), measured at treatment entry and discharge, and postdischarge follow-up. Age of depression onset, suicidality, self-injury, and childhood trauma also were assessed at treatment entry.

**Results:**

Of 200 adolescent participants who completed treatment entry and discharge assessments, the mean (SD) age was 16.2 (1.5) years; 109 reported being assigned female at birth (54.5%), 35 were TGD (17.5%), and 66 (49.3%) completed 1-month follow-up. TGD participants had an earlier mean (SD) age of depression onset (TGD: 10.8 [2.4] years vs cisgender: 11.9 [2.3] years; difference: 1.07 years; 95% CI, 0.14-2.01 years; *P* = .02), higher mean (SD) suicidality scores (TGD: 44.4 [23.1] vs cisgender: 28.5 [25.4]; difference: 16.0; 95% CI, 6.4-25.5; *P* = .001), more self-injurious behavior (mean [SD] RBQ-A score for TGD: 3.1 [2.5] vs cisgender: 1.7 [1.9]; difference: 1.42; 95% CI, 0.69-2.21; *P* = .001) and more childhood trauma (eg, mean [SD] CTQ-SF score for emotional abuse in TGD: 12.7 [5.4] vs cisgender: 9.8 [4.7]; difference: 2.85; 95% CI, 1.06-4.64; *P* = .002). The TGD group also had higher symptom scores (CES-D mean difference: 7.69; 95% CI, 3.30 to 12.08; *P* < .001; MASC mean difference: 7.56; 95% CI, 0.46 to 14.66; *P* = .04; and DERS mean difference: 18.43; 95% CI, 8.39 to 28.47; *P* < .001). Symptom scores were significantly higher at entry vs discharge (CES-D mean difference, −12.16; 95% CI, −14.50 to −9.80; *P* < .001; MASC mean difference: −3.79; 95% CI, −6.16 to −1.42; *P* = .02; and DERS mean difference: −6.37; 95% CI, −10.80 to −1.94; *P* = .05) and follow-up (CES-D mean difference: −9.69; 95% CI, −13.0 to −6.42; *P* < .001; MASC mean difference: −6.92; 95% CI, −10.25 to −3.59; *P* < .001; and DERS mean difference: −12.47; 95% CI, −18.68 to −6.26; *P* < .001).

**Conclusions and Relevance:**

This cohort study found mental health disparities in TGD youth relative to cisgender youth, with worse scores observed across assessment time points. For all participants, primary clinical outcome measures were significantly lower at treatment discharge than at entry, with no significant differences between discharge and 1-month follow-up. Given the substantial degree of mental health disparities reported in TGD individuals, these findings warrant focused clinical attention to optimize treatment outcomes in gender minority populations.

## Key Points

**Question:**

Do transgender and gender diverse adolescents enrolled in acute residential treatment enter the program with worse psychiatric symptoms and do they remain worse after treatment, relative to cisgender adolescents?

**Findings:**

In this cohort study of 200 adolescents who participated in a 2-week acute residential treatment program for psychiatric disorders, the transgender and gender diverse group exhibited significantly worse symptoms at treatment entry relative to the cisgender group. Both groups had fewer depressive and anxiety symptoms and less emotional dysregulation at treatment discharge.

**Meaning:**

These findings suggest that clinical attention should focus on mental health disparities in transgender and gender diverse youth to optimize treatment approaches for this minority population.

## Teaser

This cohort study examines mental health outcomes before and after acute residential psychiatric treatment among adolescents who are transgender and gender diverse vs cisgender.

## Introduction

Transgender and gender diverse (TGD) individuals, also referred to as gender minorities, have a gender identity that differs from their sex assigned at birth (ie, natal sex). Although significantly more data on estimates of adults identifying as TGD are available,^1,2^ TGD adolescents (aged 13 to 17 years) are estimated to make up approximately 0.7% of the US population (approximately 150 000 youth).^3^ Furthermore, in the Minnesota Student Survey, of 80 929 students in 9th and 11th grade asked about gender identity, 2185 students (2.7%) identified as TGD.^4^ Although the field of gender identity within pediatrics is evolving, research in most areas related to treatments for TGD youth remains limited,^5^ with most of the existing research in this population being primarily derived from school-based studies.

However, there is evidence that TGD youth face substantial health disparities relative to cisgender peers (ie, those matching in natal sex and gender identity).^4,6,7,8,9,10^ In a recent study, 33.7% of youth identifying as transgender or nonbinary endorsed suicidal ideation compared with 18.9% of nontransgender peers (sex assigned at birth not asked, thus the term cisgender was not used).^8^ A 2017 population-based study of US high school students found similar disparities, with 43.9% of TGD youth reporting that they had considered suicide (compared with cisgender male: 11.0%; cisgender female: 20.3%) and 34.6% having attempted suicide in the past year (compared with cisgender male: 5.5%; cisgender female: 9.1%).^11^ TGD adolescents are vulnerable to facing increased challenges relative to cisgender peers due to experiences of minority stress, such as bullying, and also experience higher rates of mental health problems, such as depression and anxiety.^11,12,13^

In addition to high rates of mental health conditions among TGD individuals, there is a problematic treatment gap for gender minority groups. A systematic review indicated that TGD individuals face several barriers to accessing mental health care, including concerns that they will be pathologized or stereotyped because of their gender identity, concerns that clinicians will be uninformed or unsupportive, and financial barriers.^14^ In a 2017 study of TGD youth,^15^ participants reported at least one “transnegative” experience during mental health treatment; these experiences included working with counselors who lacked basic knowledge about TGD issues such as lack of access to public restrooms and lack of protections from hate crimes. Transnegative experiences also included invalidating interactions, such as when clinicians repeatedly misgender patients (eg, using the incorrect name or pronouns) or assume that a TGD identity is an indicator of pathology. After being invalidated by a mental health professional, patients reported feeling less motivated to reach out to their clinician for treatment. Importantly, participants also reported at least one “transaffirmative” experience as well, including a valued therapeutic alliance and advocacy by the mental health clinician on the patient’s behalf,^15^ which is consistent with data showing that psychotherapy can also include positive, validating experiences for TGD individuals.

Given high rates of mental illness within TGD youth, due in part to underlying vulnerabilities associated with gender minority stress-related stigma,^12,16,17^ this study examined mental health within TGD youth admitted to acute residential treatment (ART). TGD youth were compared with cisgender peers at treatment entry and discharge. A subset of youth completed remote assessments at 1-month postdischarge. It was hypothesized that severity of psychiatric symptoms at treatment entry would be significantly worse in TGD vs cisgender participants. Furthermore, although participants were anticipated to demonstrate less severe depressive and anxiety symptoms and emotion dysregulation at discharge, it was hypothesized that treatment-associated differences would be of a lesser magnitude in TGD relative to cisgender participants. Observation within clinical settings provides an opportunity to study complex psychopathology among youth and can inform treatment interventions focused on unique needs of TGD youth. Given that many TGD youth do not have access to specialized mental health care services, it is of critical importance to understand whether this population is well-served by a general psychiatric treatment model.

## Methods

### Participants and Procedures

In this cohort study, participants were adolescents aged 13 to 17 years and admitted into a short-term acute residential treatment (ART) program for approximately 2 weeks. Participants were asked to provide their sex assigned at birth and select all gender identities that applied: cisgender (girl or boy); TGD (transgender boy, transgender girl, gender queer or nonconforming, other or none of the above, or multiple gender identities). Those reporting that their sex assigned at birth aligned with their gender identity comprised the cisgender group and those reporting that their sex assigned at birth was not aligned with their gender identity comprised the TGD group. Given mental health differences among racial groups, as well as effects of racial discrimination on mental health in adolescents, race information was collected at treatment entry. Participants were presented with the following options and asked to choose all that described their race/ethnicity: White, African American/Black, Alaskan Native/American Indian, Latinx/Hispanic, Asian/Pacific Islander, Multiracial, or none of the above (other). Participants meeting criteria for substance use disorders were excluded (alcohol and marijuana use permitted), as the prevalence of substance use disorders was negligible in the TGD group.

All adolescents entering the ART program provided oral assent to participate in the quality improvement (QI) protocol, approved by the Massachusetts General Brigham institutional review board. Parents or guardians were contacted at time of assent to provide oral consent for QI protocol participation, which all ART program participants completed. All data were deidentified prior to data analyses. The QI protocol included a structured clinical diagnostic interview and completion of surveys (via Research Electronic Data Capture), within 48 hours after admission and 48 hours before discharge. QI study measures were collected under staff supervision and adolescents could withdraw any time; no adolescents withdrew. A voluntary 1-month remote follow-up was added to the QI protocol after the study began, thus only a portion of participants had an opportunity to complete follow-up assessment. This study followed the Strengthening the Reporting of Observational Studies in Epidemiology (STROBE) reporting guideline.

### Measures

Clinical assessments were conducted at treatment entry, including age of onset of depression, suicidality (past month and lifetime), self-injury, and childhood trauma. The Mini International Neuropsychiatric Interview Kid Screen (MINI-KID),^18^ a structured clinical interview, was used to determine psychiatric diagnoses based on the *Diagnostic and Statistical Manual of Mental Disorders* (Fourth Edition) (*DSM-IV*).^19^ Age of onset of first depressive episode and suicidality scores were also determined from the MINI-KID, administered by a trained staff member at treatment entry.

Clinical outcome measures examined included (1) the Risky Behavior Questionnaire for Adolescents (RBQ-A)^20^ self-injury: scale 0 to 4, subscale score range 0 to 8, higher scores reflect more self-injurious behavior; (2) the Childhood Trauma Questionnaire Short-Form (CTQ-SF)^21^: scale 1 to 5, score range 5 to 25, higher scores reflect more endorsement of childhood neglect and abuse; (3) the Center for Epidemiologic Studies Depression Scale (CES-D)^22^: scale 0 to 3, score range 0 to 60, higher scores reflect more depressive symptoms; (4) the Multidimensional Anxiety Scale for Children (MASC)^23^: scale 0 to 3, score range 0 to 117, higher scores reflect more anxiety; and (5) the Difficulties in Emotion Regulation Scale (DERS)^24^: scale 1 to 5, score range 36 to 180, higher scores reflect more emotion dysregulation.

### Statistical Analysis

Statistical analyses were conducted using SPSS version 24.0 (IBM Corp) from October 2019 to March 2021. Data were examined for outliers and coding errors, and for normality using skewness and kurtosis (skewness: −0.653 to 0.469; kurtosis: −1.0 to 1.5). χ^2^ tests were used to test for group differences in racial/ethnicity representation and prevalence of clinical diagnoses. For primary hypotheses, TGD and cisgender were comparator groups, tested to determine if clinical symptoms were greater at entry in the TGD vs cisgender group and whether clinical symptoms changed significantly across assessment time points. Secondary analyses tested for interactions between group and assessment time points. Linear mixed model analyses were performed with group (TGD, cisgender) and assessment time point (entry, discharge, 1-month follow-up) as fixed factors and participant as a random factor. Age was included as a covariate. Post hoc analyses were performed to examine significant main effects or interactions. Two-tailed *t* tests were used, and the significance threshold was *P* < .05.

## Results

### Demographic Information

Of 200 adolescent participants who completed treatment entry and discharge assessments, 109 reported being female (55%) and 91 reported being male (45%) assigned at birth; 35 adolescents identified as TGD (17.5%) and identified as transgender boys (n = 9), transgender girls (n = 2), gender nonconforming or gender queer (n = 22), another gender identity (n = 9), or endorsed more than one identity (n = 7); 165 adolescents identified as cisgender (82.5%) and as cisgender girls (n = 76; 38%) or cisgender boys (n = 89; 45%); 66 (49.3%) completed follow-up data; 183 adolescents were White (91.5%); and the mean (SD) age was 16.2 (1.5) years. Demographic characteristics, presence of clinical diagnoses (eg, major depressive disorder), treatment entry clinical measures, and clinical outcome data (eg, MASC) for the group with follow-up data compared with the group without follow-up data are presented in eAppendix 1, eTable 1, eTable 2, and eTable 3 in the Supplement.

Mean (SD) age differed significantly between TGD (15.5 [1.5] years) and cisgender (16.4 [1.5] years) (mean difference, 0.82; 95% CI, 0.28-1.35; *P* = .003) groups. Treatment duration did not differ between groups (TGD: 15.3 [3.3] days; cisgender: 16.6 [8.1] days, *P* = .40), thus it was not a covariate. TGD and cisgender groups did not differ on racial or ethnic distribution (Table 1).

**Table 1.** Participant Ethnic and Racial Identity

Ethnic and racial identity	Participants, No. (%)		
	TGD	Cisgender	Overall
White	33 (94.3)	151 (90.9)	183 (91.5)
African American/Black	1 (2.9)	2 (1.2)	3 (1.5)
Alaskan Native/American Indian	0	1 (0.6)	1 (0.5)
Asian/Pacific Islander	0	3 (1.8)	3 (1.5)
Multiracial	1 (2.9)	3 (1.8)	4 (2.0)
Latinx/Hispanic	2 (5.7)	7 (4.2)	9 (4.5)
Other race/ethnicity	3 (8.6)	6 (3.6)	9 (4.5)

Abbreviation: TGD, transgender and gender diverse.

### Treatment Entry: Clinical Diagnostic and Outcome Measures

The TGD group had significantly higher prevalence of clinical diagnoses at treatment entry: major depressive disorder (TGD: 84.4% [27 participants] vs cisgender: 64.4% [98 participants]; χ^2^ = 4.81; *P* = .03), social phobia (TGD: 60.0% [18 participants] vs cisgender: 33.3% [53 participants]; χ^2^ = 7.65; *P* = .006), obsessive compulsive disorder (TGD: 12.5% [4 participants] vs cisgender: 3.3% [6 participants]; χ^2^ = 4.32; *P* = .04) and PTSD (TGD: 25.8% [8 participants] vs cisgender: 12.2% [20 participants]; χ^2^ = 3.93; *P* = .04) (Table 2). The TGD group also presented with earlier mean (SD) age of depression onset (TGD: 10.8 [2.4] years vs cisgender: 11.9 [2.3] years; difference: 1.07 years; 95% CI, 0.14-2.01 years; *P* = .02), higher mean (SD) suicidality scores (TGD: 44.4 [23.1] vs cisgender: 28.5 [25.4]; difference: 16.0; 95% CI, 6.4-25.5; *P* = .001), more self-injurious behavior (mean [SD] RBQ-A score for TGD: 3.1 [2.5] vs cisgender: 1.7 [1.9]; difference: 1.42; 95% CI, 0.69-2.21; *P* = .001), and more childhood trauma (eg, mean [SD] CTQ-SF score for emotional abuse in TGD: 12.7 [5.4] vs cisgender: 9.8 [4.7]; difference: 2.85; 95% CI, 1.06-4.64; *P* = .002) (Table 3).

**Table 2.** DSM-IV MINI-KID Psychiatric Diagnoses at Treatment Entry

Diagnoses	Participants, No. (%)			χ^2^	*P* value
	TGD	Cisgender	Overall		
Major depressive disorder	27 (84.4)	98 (64.5)	125 (67.9)	4.81	.03
Bipolar disorder					
I	4 (12.9)	9 (5.5)	13 (6.6)	2.34	.13
II	0	3 (1.8)	3 (1.5)	0.57	.45
Generalized anxiety disorder	20 (62.5)	74 (45.1)	94 (48.0)	3.24	.07
Panic disorder	7 (24.1)	21 (13.6)	28 (15.3)	2.08	.15
Social phobia (generalized)	18 (60.0)	53 (33.3)	71 (37.6)	7.65	.006
Obsessive compulsive disorder	4 (12.5)	6 (3.7)	10 (5.1)	4.32	.04
Posttraumatic stress disorder	8 (25.8)	20 (12.2)	28 (14.4)	3.93	.047
ADHD (combined)	6 (18.8)	18 (11.0)	24 (12.2)	1.51	.22
Eating disorder					
Anorexia	2 (6.3)	8 (4.9)	10 (5.1)	0.10	.75
Bulimia	2 (6.3)	10 (6.1)	12 (6.1)	0.00	.97
Co-occurring diagnoses	27 (84.4)	148 (89.7)	175 (88.8)	0.77	.38

Abbreviations: ADHD, attention-deficit/hyperactivity disorder; TGD, transgender and gender diverse.

**Table 3.** Treatment Entry: Depression Onset, Suicidality, Self-injury, and Childhood Trauma

Clinical measure	Mean (SD)		*P* value
	TGD	Cisgender	
Age of depression onset, y	10.8 (2.4)	11.9 (2.3)	.02
Suicidality score	44.4 (23.1)	28.5 (25.4)	.001
RBQ-A			
Self-injurious behavior	3.1 (2.5)	1.7 (1.9)	.001
CTQ-SF			
Total score	49.4 (17.4)	40.6 (11.5)	<.001
Emotional abuse	12.7 (5.4)	9.8 (4.7)	.002
Emotional neglect	13.3 (4.6)	11.7 (5.1)	.08
Physical abuse	6.8 (4.3)	5.7 (1.6)	.02
Physical neglect	8.6 (2.4)	7.3 (2.3)	.005
Sexual abuse	8.1 (6.2)	6.0 (3.1)	.005

Abbreviations: CTQ-SF, Childhood Trauma Questionnaire Short-Form; RBQ-A, Risky Behavior Questionnaire for Adolescents; TGD, transgender and gender diverse.

### Group and Assessment Time Points: Changes in Clinical Outcome Measures

There was a main effect of group for each measure, with the TGD group exhibiting significantly higher scores than the cisgender group, collapsed across assessment time points: CES-D (Figure A) (difference: 7.69; 95% CI, 3.30 to 12.08; *P* < .001), MASC (Figure B) (difference: 7.56; 95% CI, 0.46 to 14.66; *P* = .04), and DERS (Figure C) (difference: 18.43; 95% CI, 8.39 to 28.47; *P* < .001). There also was a main effect of time for each symptom, with significantly lower scores over time points, collapsed across group. Post hoc analyses showed that scores were significantly different between entry and discharge (CES-D mean difference: −12.16; 95% CI, −14.5 to −9.80; *P* < .001; MASC mean difference: −3.79; 95% CI, −6.16 to −1.42; *P* = .02; DERS mean difference: −6.37; 95% CI, −10.80 to −1.94; *P* = .05) and entry and follow-up (CES-D mean difference: −9.69; 95% CI, −13.0 to −6.42; *P* < .001; MASC mean difference: −6.92; 95% CI, −10.25 to −3.59; *P* < .001; DERS mean difference: −12.47; 95% CI, −18.68 to −6.26; *P* < .001), but not between discharge and follow-up (CES-D mean difference: 2.48; 95% CI, 0.83 to 5.79, *P* = .14; MASC, −3.14; 95% CI, −6.47 to 0.19; *P* = .07; DERS mean difference: −6.10; 95% CI, −12.35 to 0.15; *P* = .06). There were no significant group × time point interactions for CES-D (*P* = .25), MASC (*P* = .16) or DERS (*P* = .65). See eAppendix 2 in the Supplement for comparisons of groups with and without follow-up data.

**Figure.**
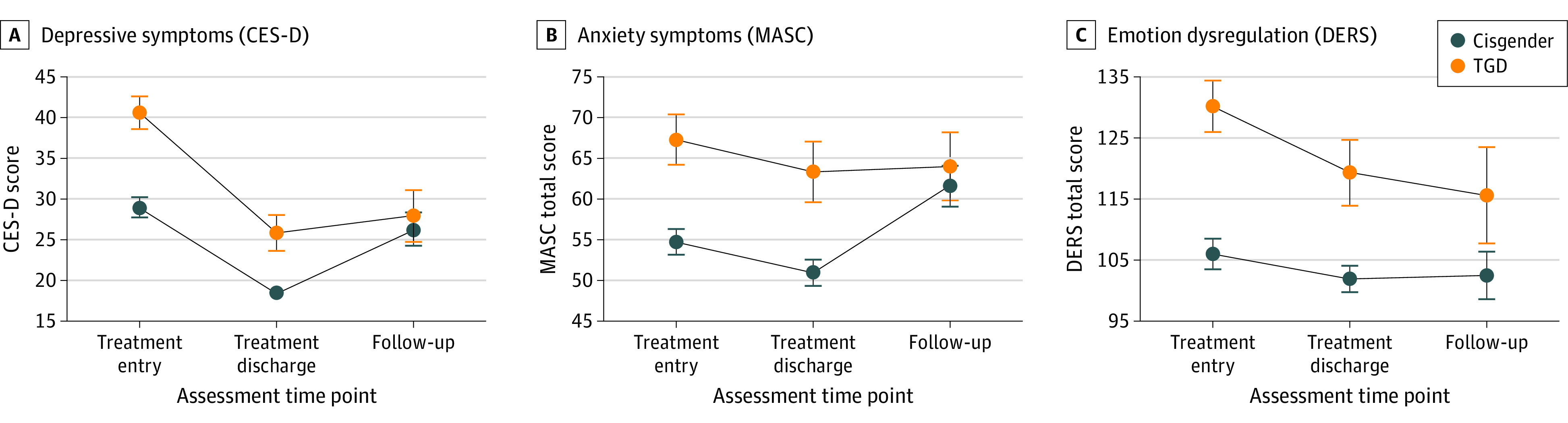
Changes in Mental Health Symptoms Over Time From Treatment Entry to Treatment Discharge to Follow-up Data represent mean scores and reflect main effects of group and assessment time point. Error bars denote standard error of the mean. CES-D indicates Center for Epidemiologic Studies Depression Scale; MASC, Multidimensional Anxiety Scale for Children; DERS, Difficulties in Emotion Regulation Scale; TGD, transgender and gender diverse.

## Discussion

Previous studies have examined mental health disparities between TGD and cisgender youth^12,25^; however, to our knowledge, the present study is the first to examine TGD mental health during and following short-term, insurance-based acute residential treatment. In the present study, adolescent TGD participants were evaluated relative to cisgender participants at treatment entry and discharge. Although both TGD and cisgender adolescents were found to have significantly lower depressive and anxiety symptom scores and less emotional dysregulation over the course of treatment, TGD adolescents maintained significantly greater severity of psychiatric symptoms relative to cisgender peers across assessment time points. Participants in the TGD group presented to treatment with an earlier age of onset of depression, more severe suicidality, more self-injurious behaviors, and more childhood trauma, as well as greater depressive and anxiety symptoms, and worse emotional regulation. These findings are consistent with previous studies reporting more severe mental health symptoms and more maladaptive behaviors, such as suicidal and self-injurious behaviors, in TGD adolescents compared with cisgender adolescents.^25,26^ The current study expands upon previous research to include characterization of depressive symptoms, anxiety symptoms, and emotional dysregulation, and the association of mental health treatment with these psychiatric symptoms, in TGD adolescents seeking treatment at an acute residential level of care.

Several studies have found that discrimination and a lack of knowledge by clinicians create major barriers for TGD individuals seeking care, thereby contributing to greater stress and health disparities in this population.^27^ Although TGD youth may present to treatment with unique needs, such as familial rejection and challenges with school policies,^15,25,28,29^ they may not have access to specialized services for gender minorities, due in part to geographic and/or financial limitations. Consequently, it is critically important to evaluate insurance-based programs that provide access to a broader population of TGD youth. The results of the present study provide a promising indication that insurance-based programs can benefit both TGD and cisgender youth.

Adolescents enrolled in the ART program engaged in milieu-based treatment, comprised of cognitive behavioral therapy, dialectical behavioral therapy, motivational interviewing, and individual, group, and family therapy. Participants also were assessed by a psychiatrist twice a week, and pharmacologic interventions were introduced as needed. Although it is not possible to determine which aspects of care may have contributed to better mental health outcomes, these preliminary data provide important foundational evidence that TGD youth can benefit from acute residential treatment. Practices that promote empathy, alliance, positive regard and acceptance, and social transitioning among TGD youth (eg, chosen name and pronouns, access to restrooms consistent with gender identity and presentation^30^) positively impact psychotherapy outcomes for TGD individuals.^15,27,31^ Future studies may choose to consider how to best optimize treatment for TGD youth by evaluating the relative effectiveness of different treatment interventions and modalities. Efficacious elements of treatment that could benefit TGD youth could be implemented in schools and community centers through psychoeducation, emotion regulation skills training, and mindfulness for TGD youth who do not readily have access to psychiatric care. This study is an important initial step in identifying potential treatment modalities for TGD adolescents.

In addition, as TGD individuals are not a homogeneous group, all individuals who identify as TGD might not benefit from the same care.^32^ Thus, further research should consider the needs of TGD youth with diverse identities. In this sample, TGD individuals were twice as likely to identify as nonbinary or another gender identity than to identify with a binary transgender identity (transgender boy or transgender girl). This identity distribution is consistent with limited previous data regarding the prevalence of individuals who identify as other or nonbinary relative to individuals who identify as (binary) transgender,^33^ particularly at younger ages.^9^ Thus, demographic estimates that limit gender identity to cisgender and (binary) transgender underestimate proportions of TGD individuals who represent binary and nonbinary gender identities. Consequently, current estimates and psychiatric symptoms in TGD populations likely miss critical information. Thus, more specific and detailed assessments of gender identity in demographic surveys and in clinically and empirically based investigations are needed. Such assessments would more accurately be representative and could help elucidate aspects of mental health care that would benefit individuals who are neither cisgender nor (binary) transgender.

### Limitations

This study has limitations that must be considered. Beyond demographic assessment of self-identification at treatment entry, there were no assessment measures specifically querying dimensions of gender, such as felt gender, gender noncontentedness, and gender nonconformity.^34^ Furthermore, there was insufficient statistical power to compare subgroups of TGD or cisgender participants in the present investigation. In addition to the need for more inclusive gender identifying questions, the clinical inventories used in this study may not comprehensively capture the complexities of interactions between gender identity, gender minority stress, and psychiatric symptoms in TGD youth. Measures from the Gender Minority Stress and Resilience (GMSR),^35,36^ which have been validated for adults but not yet for youth, might elucidate such complex associations between identity and mental health. Additionally, the Parental Attitudes of Gender Expansiveness Scale for Youth (PAGES-Y)^37^ is a promising acceptance scale that could provide useful information about individuals’ familial context.

Furthermore, the participants in this study were predominately White and non-Hispanic, which is not representative of the racial and ethnic diversity of TGD youth in the United States.^9^ This is important to note as the intersection of gender and racial/ethnic identity may present unique challenges.^15^ Future research should focus on the experiences of TGD youth of color to better understand the interaction of these identities and how this might impact treatment outcomes.^38^

An additional limitation was that participants were only included if families were engaged in treatment, as the ART program requires family participation, regardless of family acceptance or awareness of their child’s gender identity. Acceptance and rejection play critical roles in the psychosocial health of TGD individuals,^29^ thus environmental factors are critical to consider, especially as youth return to affirming or nonaffirming home and school environments following treatment. Finally, this study did not have a nontreatment comparison group. Although gains are likely to have been due to treatment, it is plausible that symptoms could have remitted in adolescents with time; for example, on a waiting list. Despite these limitations, these findings offer important insights into TGD adolescents’ mental health needs and treatment response and may be useful in informing future approaches to treating TGD youth.

Treatment-related improvements and maintaining gains in functioning after discharge, while preventing reintensification of symptoms and reengagement in maladaptive behaviors, are of critical concern for all youth who have undergone mental health treatment. As this was an observational study, causation underlying changes in clinical outcome measures, fewer depressive symptoms and less anxiety and emotional dysregulation, across assessments, cannot be inferred. Nonetheless, a remarkable aspect of these findings is that the subset of TGD participants who completed postdischarge follow-up maintained less symptoms 1-month post treatment. Although there were no significant group × assessment time point interactions, TGD participants did not differ significantly from the cisgender group when scores were averaged across time points. These follow-up data are encouraging, suggesting that improvements associated with treatment are maintained 1-month posttreatment, regardless of gender identity. It is important to recognize, however, that a lack of symptom disparity in TGD vs cisgender groups when averaged across assessments (see eAppendix 2 in the Supplement), could have reflected a response bias in those with relative to the those without follow-up data. Interestingly, those with follow-up data had greater prevalence of major depressive disorder and anxiety-related diagnoses, more endorsement of childhood maltreatment, and higher MASC scores at treatment entry and discharge. Clearly, these findings need future investigation, with focused attention on impacts of anxiety and early life adversity on posttreatment follow-up. Nonetheless, this approach reflects the utility of posttreatment assessment and the need for further research. Collection of information about environmental factors, peer relationships, family and community support, and longer-term follow-up are highly warranted to aid in identification of factors preventing posttreatment relapse.

## Conclusions

In conclusion, TGD participants presented for treatment with more severe psychiatric symptoms than cisgender youth, which is aligned with evidence of a mental health disparity between these groups. This is not to say that identifying as TGD is inherently associated with mental health difficulties. Rather, a combination of gender minority stressors, such as peer bullying and rejection, create a complex clinical picture leading to increased psychiatric risk.^16,39,40^ As the rate of TGD adolescents seeking mental health care is increasing,^26^ it is encouraging that even nonspecialized treatment (ie, those not tailored to needs of gender minority populations), such as a general, insurance-based acute residential treatment program, can be successful in reducing clinical symptoms in TGD youth. Importantly, there is a call to action for health care clinicians to more thoroughly screen and sensitively assess youth who identify as a gender minority, in an effort to provide the most effective treatment for this vulnerable psychiatric population.

## Notes

Supplement. **eAppendix 1.** Comparison of One-Month Post-Discharge in Follow-Up and No Follow-Up Groups **eAppendix 2.** Group and Assessment Timepoints: Changes in Clinical Outcome Measures **eTable 1.** DSM-IV MINI-KID Psychiatric Diagnoses **eTable 2.** Treatment Entry: Depression Onset, Suicidality, Self-Injury and Childhood Trauma **eTable 3.** Treatment Entry and Discharge: Depressive Symptoms, Anxiety, and Emotional Dysregulation
