# Supplementary material for: Clinical Outcomes Following Acute Residential Psychiatric Treatment in Transgender and Gender Diverse Adolescents
Source: JAMA Netw Open. 2021 Jun 21;4(6):e2113637. doi: 10.1001/jamanetworkopen.2021.13637 (PMC8218077; doi:10.1001/jamanetworkopen.2021.13637)
Supplement: Supplement. — eAppendix 1. Comparison of One-Month Post-Discharge in Follow-Up and No Follow-Up Groups eAppendix 2. Group and Assessment Timepoints: Changes in Clinical Outcome Measures eTable 1. DSM-IV MINI-KID Psychiatric Diagnoses eTable 2. Treatment Entry: Depression Onset, Suicidality, Self-Injury and Childhood Trauma eTable 3. Treatment Entry and Discharge: Depressive Symptoms, Anxiety, and Emotional Dysregulation [file jamanetwopen-e2113637-s001.pdf]

Supplemental Online Content

Silveri MM, Schuttenberg EM, Schmandt K, et al. Clinical outcomes following acute residential psychiatric treatment in transgender and gender diverse adolescents. *JAMA Netw Open.* 2021;4(6):e2113637. doi:10.1001/jamanetworkopen.2021.13637

eAppendix 1. Comparison of One-Month Post-Discharge in Follow-Up and No Follow-Up Groups

eAppendix 2. Group and Assessment Timepoints: Changes in Clinical Outcome Measures

eTable 1. DSM-IV MINI-KID Psychiatric Diagnoses

eTable 2. Treatment Entry: Depression Onset, Suicidality, Self-Injury and Childhood Trauma

eTable 3. Treatment Entry and Discharge: Depressive Symptoms, Anxiety, and Emotional Dysregulation

This supplemental material has been provided by the authors to give readers additional information about their work.

© 2021 Silveri MM et al. *JAMA Network Open.*

eAppendix 1. Comparison of One-Month Post-Discharge in Follow-Up and No Follow-Up Groups

Follow-up data were available for a subset of 66 participants (33.3% of the total sample); 37.1% of the TGD group and 32.1% of the cisgender group. The remaining $n = 134$ participants did not have follow-up data. Comparisons of demographics, diagnoses, treatment entry clinical measures and treatment entry and discharge clinical outcomes are in supplemental Tables 1-3. There were no significant differences in the numbers of TGD and cisgender participants, nor were there significant differences in ethnic and racial representation, in the follow-up group relative to the no follow-up group. For clinical diagnoses, the follow-up group had significantly higher prevalence rates of diagnoses of MDD and anxiety-related disorders (Supplemental Table 1), significantly more childhood trauma (Supplemental Table 2), and significantly higher MASC scores at entry and discharge in the follow-up group relative to the no follow-up group (Supplemental Table 3).

eAppendix 2. Group and Assessment Timepoints: Changes in Clinical Outcome Measures

For group and timepoint assessment of changes in clinical outcome measures, there remained significant main effects of time for each symptom score. Post hoc analyses showed that changes were significantly different between entry and discharge (CES-D, -13.83; 95% CI, -17.36 to -10.30, $p < 0.0001$; MASC, -3.84; 95% CI, -7.58 to -1.93, $p = 0.046$; DERS, -7.77; 95% CI, -15.1 to -0.44, $p = 0.040$) and entry and follow-up (CES-D, -11.23; 95% CI, -14.76 to -7.70, <0.0001;

© 2021 Silveri MM et al. *JAMA Network Open.*

MASC, -7.91; 95% CI, -11.67 to -4.15, $p < 0.0001$; DERS, -14.03; 95% CI, -21.40 to -6.66, $p = 0.0003$), but only between discharge and follow-up for MASC (-4.07; 95% CI, -7.83 to -0.31, $p = 0.036$), not CES-D (2.60; 95% CI, -0.93 to 6.13, $p = 0.15$) or DERS (-6.25; 95% CI, -13.62 to 1.12, $p = 0.10$). There were no main effects of group for CES-D ($p = 0.33$), MASC ($p = 0.19$) or DERS ($p = 0.17$) or any interactions between group x assessment timepoint for CES-D ($p = 0.67$), MASC ($p = 0.19$) or DERS ($p = 0.94$).

© 2021 Silveri MM et al. *JAMA Network Open.*

eTable 1. DSM-IV MINI-KID Psychiatric Diagnoses

Diagnoses	Follow-Up	No Follow-Up	Overall	χ^2
Major Depressive Disorder	85.7%*	58.7%	67.9%	13.90
Bipolar Disorder (I, II)	4.9%, 0%	7.7%, 2.3%	6.6%, 1.5%	
Generalized Anxiety Disorder	72.7%	35.4%	48.0%	24.46
Panic Disorder	24.1%	11.2%	15.3%	5.12
Social Phobia (generalized)	53.1%*	29.6%	37.6%	9.99
Obsessive Compulsive Disorder	10.6%*	2.3%	5.1%	6.23
Post-Traumatic Stress Disorder	21.2%*	10.9%	14.4%	3.93
ADHD (combined)	12.1%	12.3%	12.2%	
Eating Disorder (anorexia, bulimia)	6.1%, 9.1%	4.6%, 4.6%	5.1%, 6.1%	
Co-Occurring Diagnoses	93.9%	86.3%	88.8%	

Significant group difference in prevalence of diagnosis, $p < .05$. ADHD = attention deficit hyperactivity disorder

© 2021 Silveri MM et al. *JAMA Network Open.*

eTable 2. Treatment Entry: Depression Onset, Suicidality, Self-Injury and Childhood Trauma

Clinical Measure	Follow-Up	No Follow-Up	*p*
Age of Depression Onset (years old)	11.3 ± 2.5	11.7 ± 2.2	
Suicidality Score	40.9 ± 24.6	35.1 ± 25.7	
RBQ-A			
Self-Injurious Behavior	2.1 ± 2.0	2.4 ± 0.2	
CTQ-SF			
Emotional Abuse	10.1 ± 4.1	12.3 ± 5.6	0.014*
Emotional Neglect	10.4 ± 4.6	13.4 ± 4.6	< 0.0001*
Physical Abuse	5.5 ± 1.1	6.5 ± 3.4	0.021*
Physical Neglect	6.8 ± 2.1	8.4 ± 2.5	< 0.0001*
Sexual Abuse	6.1 ± 3.1	7.7 ± 5.6	0.050*

Data represent mean ± SD. Differences in clinical diagnostic measures were significant at $p < .05$. RBQ-A = Risky Behavior Questionnaire – Adolescent; CTQ-SF = Childhood Trauma Questionnaire Short-Form

© 2021 Silveri MM et al. *JAMA Network Open.*

eTable 3. Treatment Entry and Discharge: Depressive Symptoms, Anxiety, and Emotional Dysregulation

Clinical Measure	Assessment Timepoint	Follow-Up	No Follow-Up	*p*
CES-D	Entry	37.4 ± 13.4	33.7 ± 15.2	
	Discharge	22.9 ± 11.5	21.4 ± 11.0	
MASC	Entry	67.9 ± 17.9	58.7 ± 20.1	0.007*
	Discharge	64.9 ± 18.0	53.6 ± 21.1	0.002*
DERS	Entry	120.9 ± 29.2	120.0 ± 29.2	
	Discharge	113.2 ± 29.7	111.5 ± 25.7	

Data represent means ± standard deviation (SD). Differences in clinical outcome measures were significant at $p < 0.05$. CES-D = Center for Epidemiologic Studies Depression Scale; MASC = Multidimensional Anxiety Scale for Children; DERS = Difficulties in Emotion Regulation Scale

© 2021 Silveri MM et al. *JAMA Network Open.*
